# A novel second-stage surgical strategy for severely obese patient with pancreatic neuroendocrine tumor: a case report

**DOI:** 10.1186/s40792-022-01484-9

**Published:** 2022-06-27

**Authors:** Akira Umemura, Akira Sasaki, Hiroyuki Nitta, Hirokatsu Katagiri, Shoji Kanno, Daiki Takeda, Taro Ando, Satoshi Amano, Masao Nishiya, Noriyuki Uesugi, Tamotsu Sugai

**Affiliations:** 1 Department of Surgery, Iwate Medical University, 2-1-1 Idaidori, Yahaba, Iwate 028-3695 Japan; 2 Department of Molecular Diagnostic Pathology, Iwate Medical University, 2-1-1 Idaidori, Yahaba, Iwate 028-3695 Japan

**Keywords:** Severe obesity, Laparoscopic sleeve gastrectomy, Metabolic surgery, Pancreatic neuroendocrine tumor, Laparoscopic spleen-preserving distal pancreatectomy

## Abstract

**Background:**

Severely obese patients can have other diseases requiring surgical treatment. In such patients, bariatric surgeries are considered a precursor to operations targeting the original disease for the purpose of reducing severe perioperative complications. Pancreatic ectopic fat deposition increases pancreas volume (PV) and thickness, which can worsen insulin resistance and islet β cell function. To address this problem, we present a novel two-stage surgical strategy performed on a severely obese patient with pancreatic neuroendocrine tumor (PNET) consisting of laparoscopic sleeve gastrectomy (LSG) as a metabolic surgery followed by laparoscopic spleen-preserving distal pancreatectomy (LSPDP).

**Case presentation:**

A 56-year-old man was referred to our hospital for further investigation of a pancreatic tumor. His initial body weight and body mass index (BMI) were 94.0 kg and 37.2 kg/m^2^, respectively. Contrast computed tomography revealed an enhanced tumor measuring 15 mm on the pancreatic body. The pancreas thickness and PV were 32 mm and 148 mL, respectively. An endoscopic ultrasonographic fine needle aspiration identified the tumor as PNET-G1. We first performed LSG, the patient’s body weight and BMI had decreased dramatically to 64.0 kg and 25.3 kg/m^2^ at 6 months after LSG. The pancreas thickness and PV had also decreased to 17 mm and 99 mL, respectively, with no tumor growth. Since LSG has been shown to reduce the perioperative risk factors of LSPDP, and to improve insulin resistance and recovery of islet β cell function, we performed LSPDP for PNET-G1 as a second-stage surgery. The postoperative course was unremarkable, and the patient was discharged on postoperative day 14 without symptomatic postoperative pancreatic fistula (POPF). He was followed without recurrence or type 2 diabetes (T2D) onset for 6 months after LSPDP.

**Conclusions:**

We present a novel two-stage surgical strategy for a severely obese patient with PNET, consisting of LSG as a metabolic surgery for severe obesity, followed by LSPDP after confirmation of good weight loss and metabolic effects. LSG before pancreatectomy may have a potential to reduce pancreas thickness and recovery of islet β cell function in severely obese patients, thereby reducing the risk of clinically relevant POPF and post-pancreatectomy T2D onset.

## Background

The prevalence of severe obesity in Japan has increased due to the Westernization of the diet, increased salt content of food, and lack of exercise among its residents [1]. Severely obese patients suffer from various obesity-related health disorders, such as type 2 diabetes (T2D), hypertension, dyslipidemia, obstructive sleep apnea, and metabolic associated fatty liver disease. Severely obese patients incur higher medical costs and generally have lower life expectancy than healthy people [2]. Since 2014, the laparoscopic sleeve gastrectomy (LSG) has been covered by Japan’s national health insurance system to address this worsening issue [3].

In addition, severely obese patients may have comorbidities that require surgical treatment. Some reports have described bariatric surgery as a precursor to operations targeting the original disease for the purpose of reducing severe perioperative complications [4]. Excessive visceral adipose tissue increases the of difficulty of surgery and the risk of intraoperative bleeding, and postoperative respiratory failure may be induced by excess body weight [5]. Based on these considerations, we believe that metabolic surgery is a visible option if the originally targeted disease is treatable.

Ectopic fat deposition in the pancreas of severely obese patients has been reported [6]. Pancreatic ectopic fat may worsen insulin resistance and induce T2D due to exhaustion of insulin secretion by islet β cells. We previously reported that LSG reduces the volume of pancreatic ectopic fat and pancreas volume (PV) and that insulin sensitivity and secretion dramatically improved in T2D patients with sufficient PV reduction [7]. Therefore, we consider that LSG before pancreatectomy can reduce the risk of postoperative pancreatic fistula (POPF) and maintain islet β cell function after pancreatectomy.

In this report, we present a novel two-stage surgical strategy for a severely obese patient with pancreatic neuroendocrine tumor (PNET), namely, LSG as a metabolic surgery for severe obesity and laparoscopic spleen-preserving distal pancreatectomy (LSPDP) for PNET.

## Case presentation

**Fig. 1:**
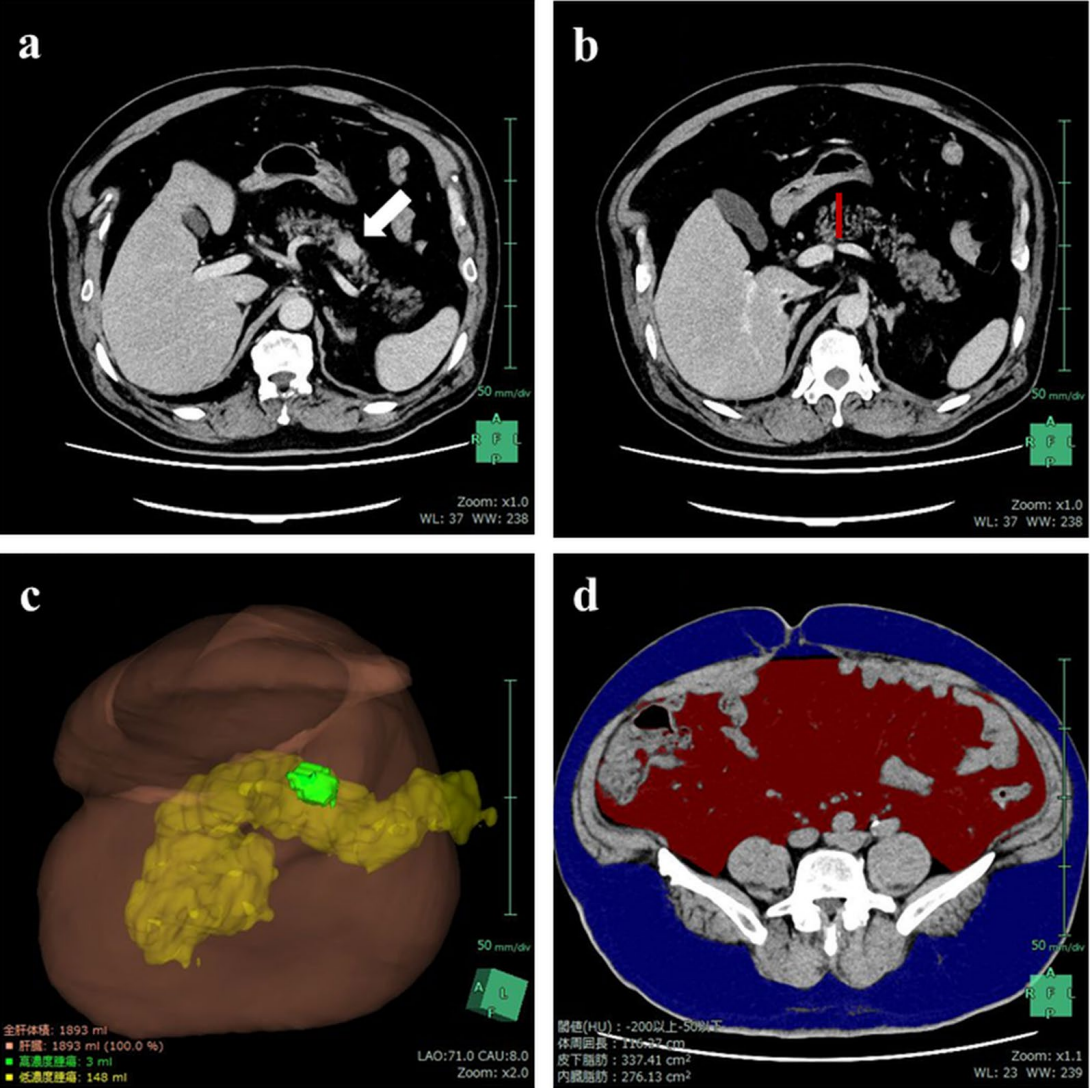
CT scan at initial visit. **a** Enhanced tumor measuring 15 mm on the pancreatic body (white arrow) and severe fat deposition were observed. **b** Thickness of the pancreas parenchyma at the bifurcation of the superior mesenteric and the splenic veins was 32 mm (red bar). **c** CT volumetry revealed that the PV was valuated as 148 ml (yellow structure). **d** Subcutaneous and visceral fat areas were 337.4 cm^2^, and 276.1 cm^2^, respectively

**Fig. 2:**
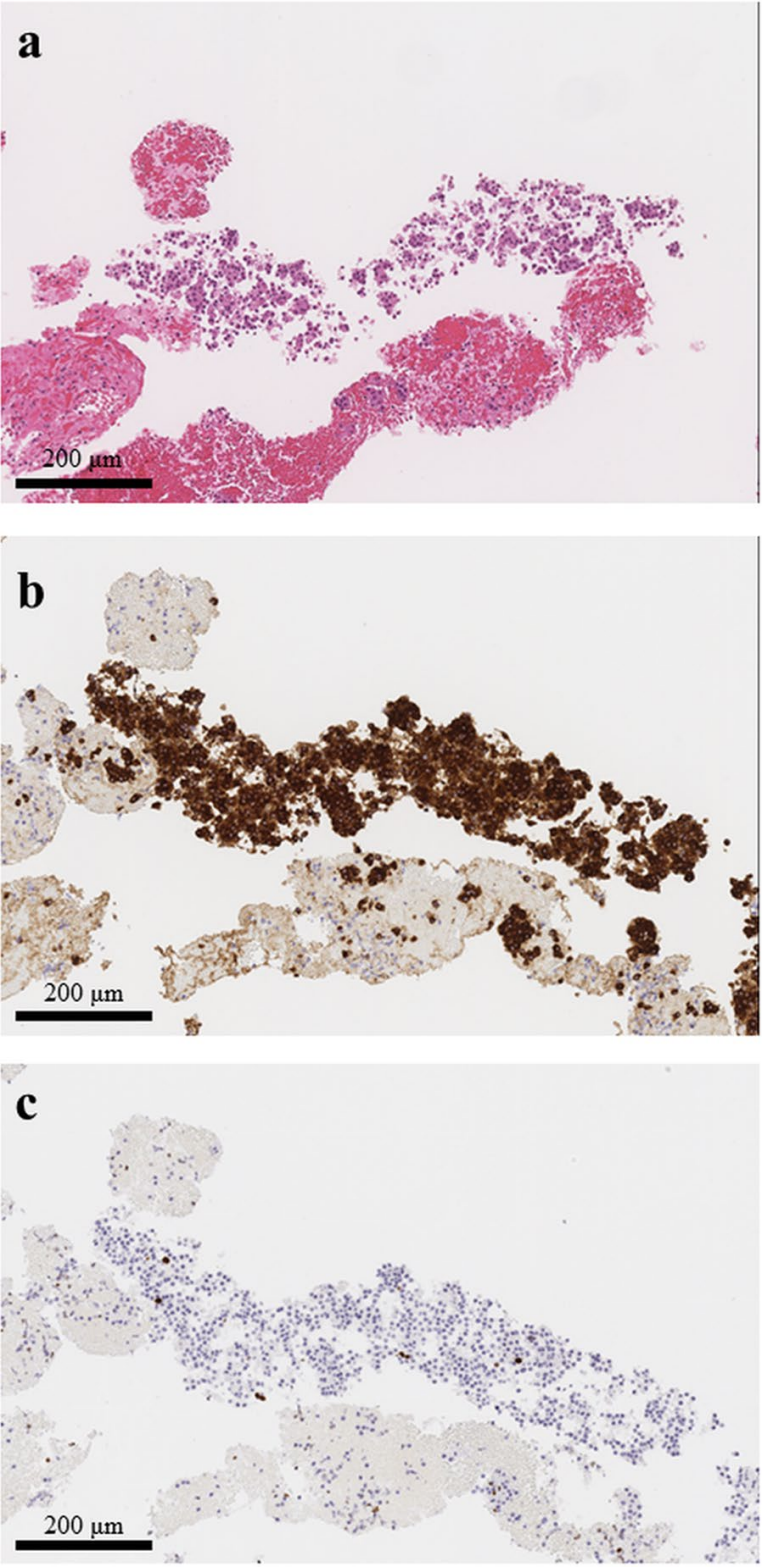
Histopathological findings of EUS-FNA. **a** Hematoxylin–eosin stain revealed that small round monotonous cells were formed into rosette-like aggregation. **b** Immunohistochemical staining showed that the tumor cells were positive for synaptophysin. **c** Ki-67 proliferation index was around 1%. All scale bars are presenting 200 μm in every picture

A 56-year-old man who had been receiving medication for hypertension for 5 years was referred to our hospital for further investigation of a pancreatic tumor detected by abdominal ultrasonography screening. His initial body weight and body mass index (BMI) were 94.0 kg and 37.2 kg/m^2^, respectively. Contrast computed tomography (CT) revealed an enhanced tumor measuring 15 mm on the pancreatic body (Fig. 1a) and severe fat deposition. The thickness of the pancreas parenchyma at the bifurcation of the superior mesenteric and splenic veins was 32 mm, and the PV was evaluated as 148 ml (Fig. 1b, c). The subcutaneous and visceral fat volumes were 337.4 cm^2^, and 276.1 cm^2^, respectively (Fig. 1d). An endoscopic ultrasonographic fine needle aspiration (EUS-FNA) revealed a rosette-like aggregation of small round monotonous cells, and immunohistochemical staining showed that the tumor cells were positive for synaptophysin (Fig. 2a, b). The Ki-67 proliferation percentage score (index) was approximately 1% (Fig. 2c). We also confirmed that every serum hormonal status of insulin, glucagon, and gastrin did not increase. Therefore, we diagnosed the pancreatic tumor as being non-functioning PNET-G1.

**Fig. 3:**
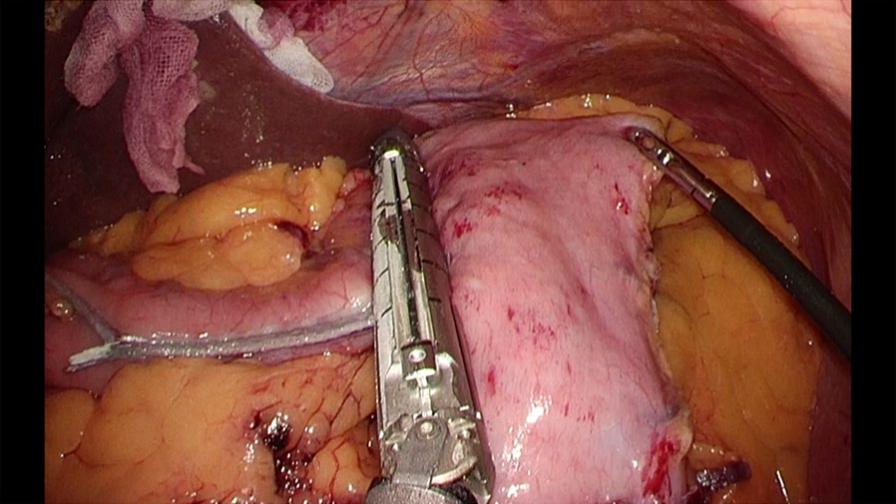
Intraoperative findings of LSG. A gastric sleeve was made by resecting the stomach alongside a 36-Fr esophagogastroduodenoscopy beginning 4 cm from the pylorus ending at the angle of His

**Fig. 4:**
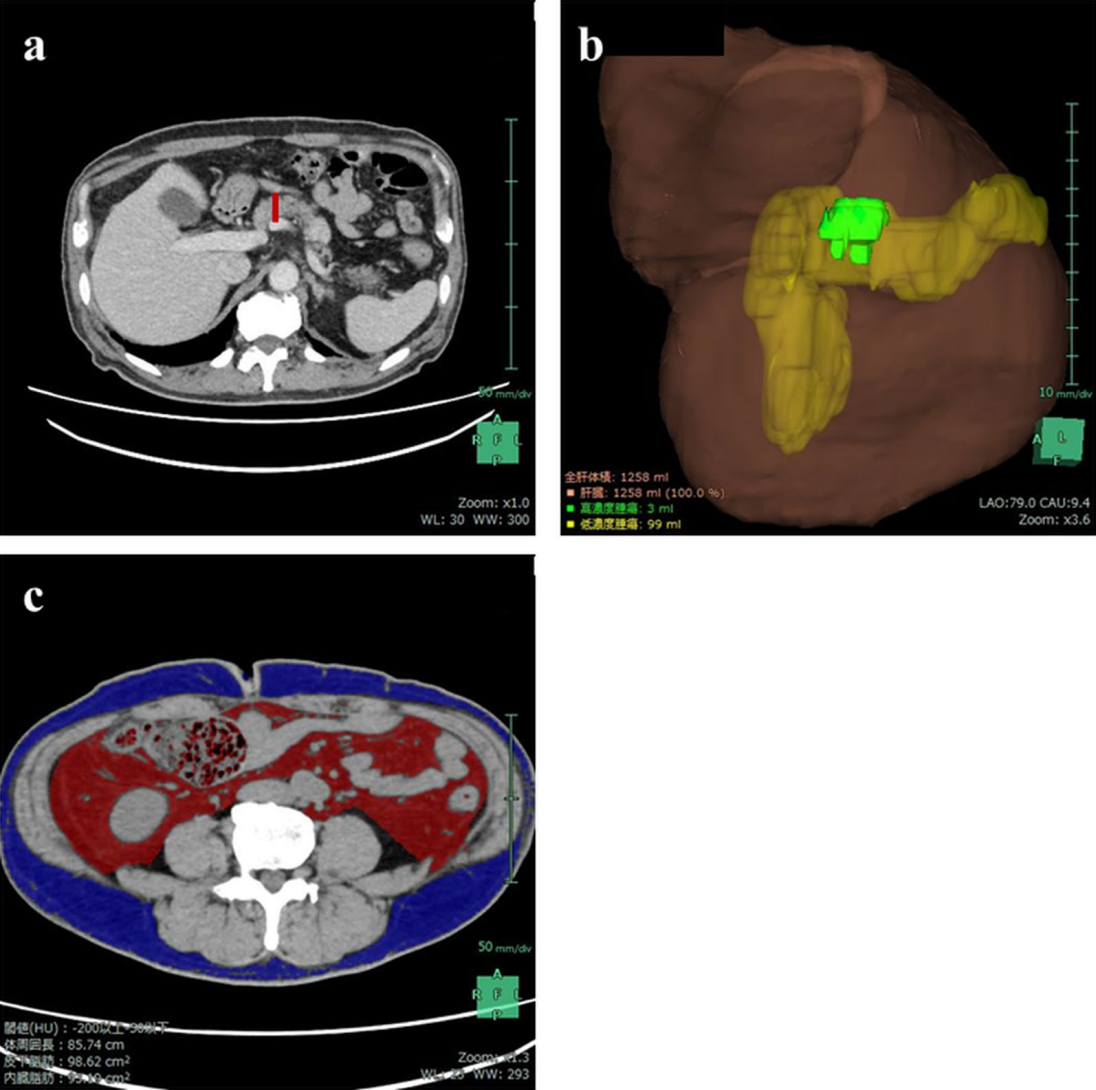
CT examination for evaluating LSG’s weight-loss and metabolic effects. **a** Pancreas parenchyma thickness decreased to 17 mm (red bar). **b** CT volumetry revealed that the PV also decreased 99 mL with no tumor growth. **c** Subcutaneous and visceral fat areas decreased to 98.6 cm^2^ and 93.2 cm^2^, respectively

Based on these examinations, we planned to perform LSG first and wait approximately 6 months after LSG to evaluate weight loss and metabolic effects before performing LSPDP for the PNET. We performed LSG, as previously reported [8] (Fig. 3) and sprayed a liquid antiadhesive agent for LSPDP (AdSpray, Terumo Corporation, Tokyo, Japan). The patient was discharged on postoperative day 5 without any perioperative complications. We followed him closely, monitoring weight loss effects and PNET size, for 6 months after LSG. His body weight and BMI decreased dramatically to 64.0 kg and 25.3 kg/m^2^, respectively. Contrast CT revealed that the pancreas parenchyma thickness and the PV also decreased to 17 mm and 99 mL, respectively, with no tumor growth (Fig. 4a, b), and the subcutaneous and visceral fat volumes decreased to 98.6 cm^2^ and 93.2 cm^2^, respectively (Fig. 4c). CT attenuations of the pancreas also improved after LSG in pancreatic head (− 28.5 HU to 37.3 HU), body (− 56.5 HU to 17.3 HU), and tail (− 58.3 HU to 1.4 HU). From these changes, pancreatic fat reduction was successfully brought by LSG. Based on these weight loss effects, we conducted that LSG had dramatically reduced the perioperative risk factors of LSPDP. Due to the improvement in his hypertension, the attending physician advised the patient he could discontinue all antihypertensive medications.

**Fig. 5:**
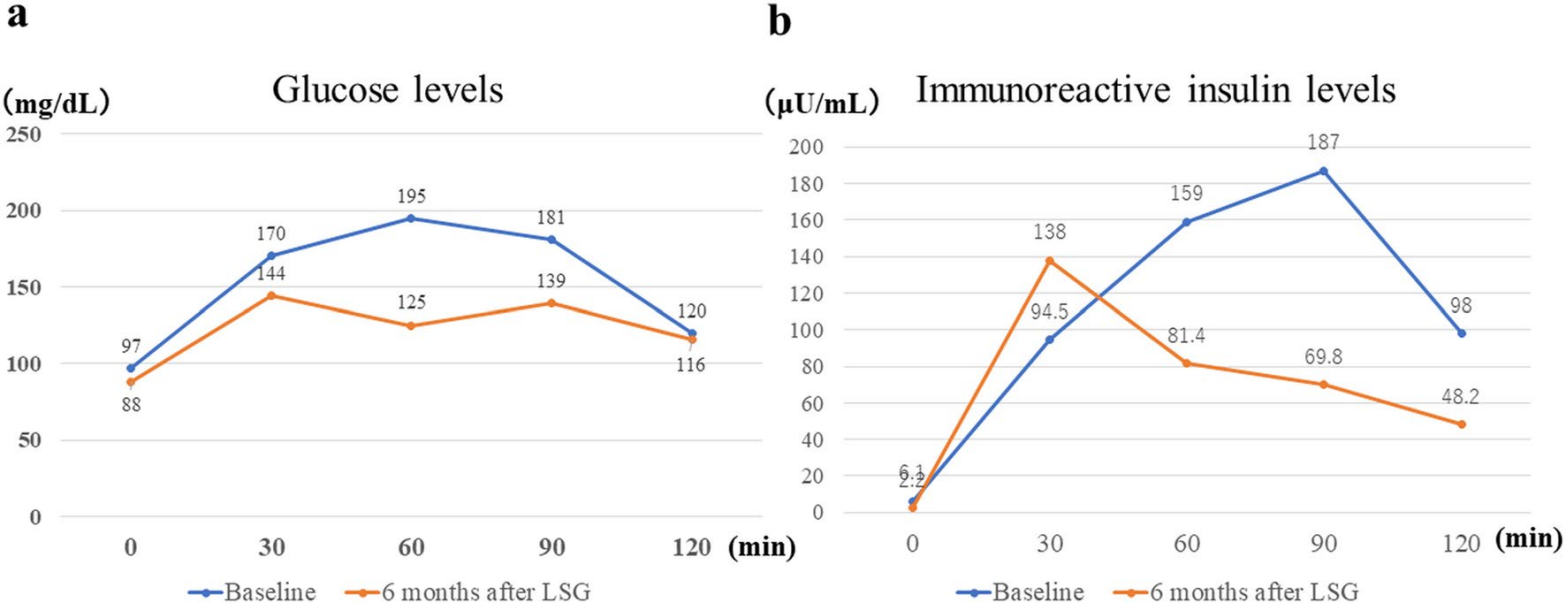
Changes in glucose and immunoreactive insulin levels using a 75-g oral glucose tolerance test at baseline and 6 months after LSG. **a** Time to peak glucose level changed from 60 to 30 min at 6 months after LSG. **b** Time to peak immunoreactive insulin level changed from 90 to 30 min at 6 months after LSG

We also evaluated metabolic effects, because the reduction of the pancreas parenchyma was the most concerning factor in relation to T2D onset after LSPDP. The results of a 75-g oral glucose tolerance test at baseline and 6 months after LSG are shown in Fig. 5. The time to peak glucose level changed from 60 to 30 min, and the time to peak immunoreactive insulin level changed from 90 to 30 min, respectively. In addition, the homeostatic model assessment of insulin resistance (2.6 to 0.5) and insulinogenic index scores (1.26 to 2.45) improved dramatically. Based on these evaluations, we confirmed a dramatic improvement in both insulin resistance and the recovery of islet β cell function. Therefore, we deemed the reduction of risk factors sufficient and decided to perform LSPDP for PNET-G1 as a second-stage surgery.

**Fig. 6:**
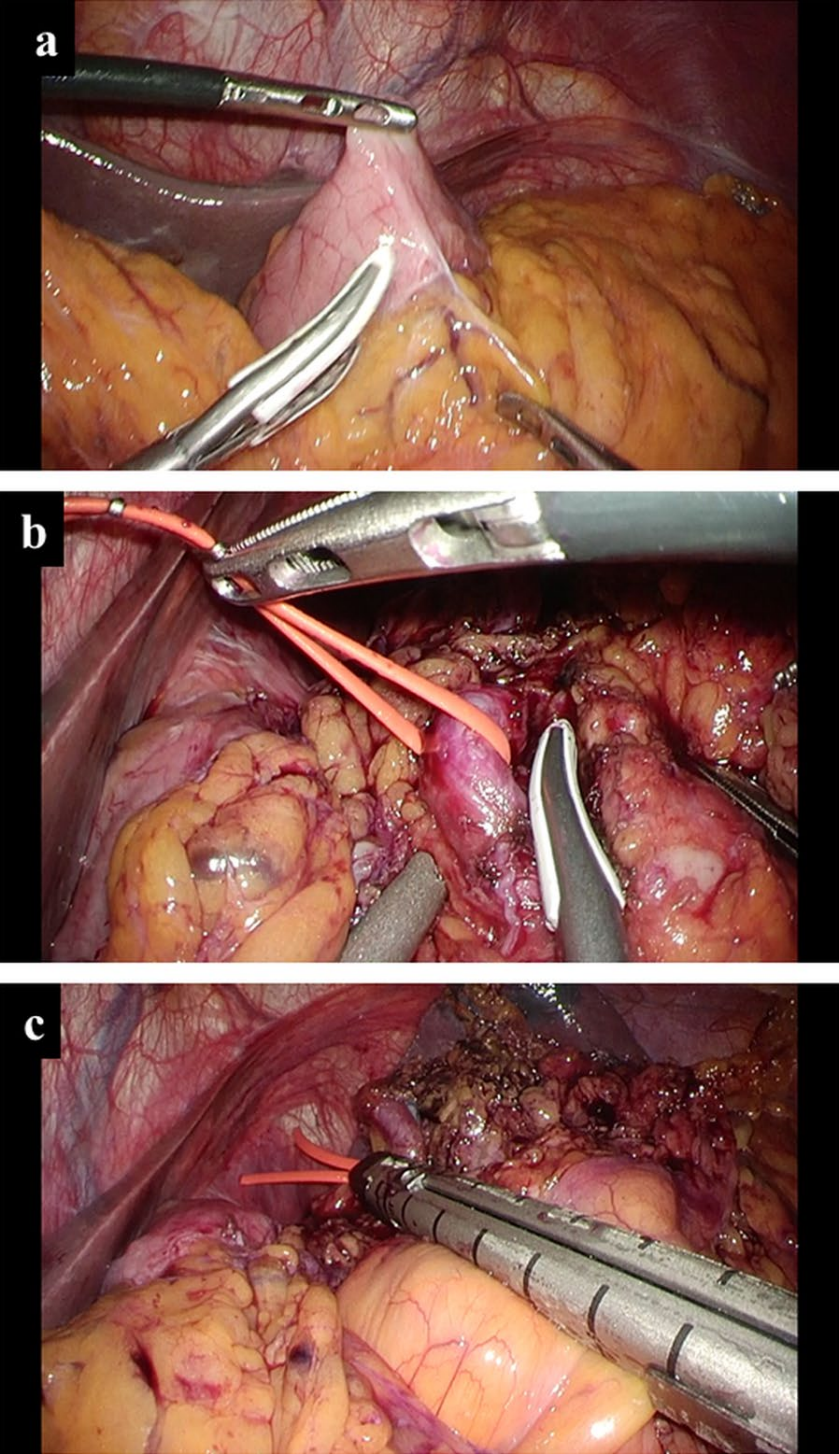
Intraoperative findings of second-stage LSPDP. **a** There were some adhesions between the gastric sleeve and the omentum due to prior LSG. **b** We taped the splenic artery and mobilized the pancreas body while transecting small branches of splenic vessels. **c** Pancreas parenchyma was transected by a linear stapler after 3-min compression

Under general anesthesia, the patient was placed in the right semi-lateral position. Carbon dioxide pneumoperitoneum pressure was set at 10 mmHg, and we inserted 4 trocars in total. For this LSPDP, we had to preserve the splenic vessels, because we had already transected the short gastric vessels during LSG. As there were some adhesions between the gastric sleeve and the omentum (Fig. 6a), we separated these adhesions and confirmed the pancreas mass. We then dissected and mobilized the caudal side of the pancreas body and tail. We dissected and taped the splenic artery at the suprapancreatic side and mobilized the pancreas body while transecting small branches of the splenic vessels (Fig. 6b). After confirming the tumor location by ultrasonography, we compressed the pancreas for 3 min and transected it using a linear stapler (Endo GIA™ 60 mm Articulating Extra Thick Reinforced Reload with Tri-Staple™ Technology, Medtronic plc, Dublin, Ireland) (Fig. 6c). The operating time and blood loss were 257 min and 70 mL, respectively.

**Fig. 7:**
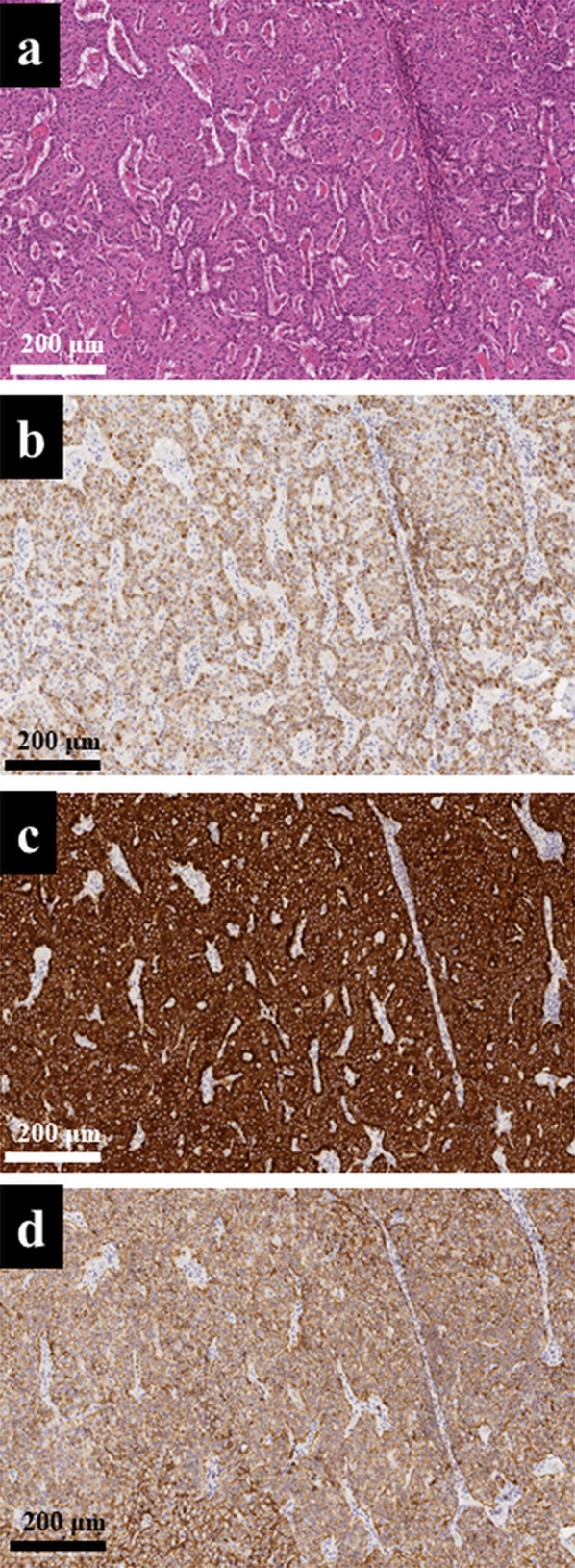
Histopathological findings of resected specimens. **a** Histopathological examination revealed that the tumor was compatible of PNET-G1 with 14 × 11 mm in size. **b** Tumor was positive for chromogranin A. **c** Tumor was positive for synaptophysin. **d** Tumor was also positive for CD56. All scale bars are presenting 200 μm in every picture

Histopathological examination revealed that the tumor was compatible with PNET-G1 being 14 × 11 mm in size on the basis of no mitosis being observed and a very low Ki-67 proliferation index (1.15%) (Fig. 7a). Immunohistochemical staining also revealed that the tumor was positive for chromogranin A, synaptophysin, and CD56 (Fig. 7b–d).

The postoperative course was unremarkable. Postoperative enhanced CT examination revealed that there were not any splenic/portal vein thrombi. The patient was discharged on postoperative day 14 without symptomatic POPF. During 6 months of the follow-up, no recurrence or T2D onset were observed after LSPDP.

## Discussion

To the best of our knowledge, this is the first case in which LSG was performed as a first-stage surgery to reduce body weight and PV with the purpose of improving islet β cell function. Bariatric surgeries, including LSG, are currently known as “metabolic surgery”, because bariatric procedures have both weight loss and metabolic effects. We applied this dynamic therapeutic mechanism to improve perioperative safety and insulin secretion after LSPDP for PNET.

In this case, we decided to perform LSG as a first-stage surgery based on histopathological findings of EUS-FNA. However, we have to know that there are some discrepancies of the Ki-67 proliferation percentage score (index) between specimens of EUS-FNA and resected specimens [9]. In this case, we diagnosed the tumor as PNET-G1 due to following reasons: (1) the tumor margin was regular, (2) the internal echo pattern was homogenous, and (3) the tumor diameter was under 20 mm [10].

Malabsorptive procedures such as laparoscopic Roux-en-Y gastric bypass (LRYGB) and laparoscopic sleeve gastrectomy with duodenojejunal bypass (LSG/DJB) are generally superior (in terms of both weight loss and metabolic effects) to restrictive procedures, such as LSG and laparoscopic adjustable gastric banding [11, 12]. However, since LRYGB is not currently covered by the Japanese national health insurance system due to the high incidence of gastric cancer in the country, Kasama et al. devised the LSG/DJB procedure [11]. Naitoh et al. reported that LSG/DJB is significantly more effective than LSG alone for patients with severe T2D and has superior weight loss effects [13]. In our patient, LSG was chosen for the following reasons: (1) the patient did not have severe T2D, and (2) LSG/DJB may have made second-stage LSPLP more complicated due to duodenojejunostomy and jejunojejunostomy.

Regarding reduction of PV, we previously reported that LSG can reduce PV in proportion to the reduction of liver volume (*r* = 0.720, *P* = 0.003), and multiple regression analyses revealed that recovery of insulin secretion had the highest correlation to pancreatic ectopic fat reduction (*β* = 0.753, *P* = 0.016, 95% confidence interval: 0.168–1.236) [7]. In addition, recent studies have reported that fat deposition in the pancreas is significantly higher in patients with prediabetes and diabetes than in healthy controls [14, 15]. Therefore, we consider that LSG as a first-stage surgery could reduce the risk of T2D onset after LSPDP. Currently, clinical importance of the qualitative evaluation of the pancreatic ectopic accumulation seems to be higher, because there have been some reports relationships between pancreatic ectopic fat accumulation and T2D, acute pancreatitis, and post-endoscopic retrograde cholangiopancreatography pancreatitis [7, 16, 17]. We previously employed measuring PV and CT values, since there are some useful modalities such as iterative decomposition of water and fat with echo asymmetry and least squares estimation magnetic resonance imaging to evaluate the pancreatic ectopic accumulation [14, 18]. In near future, further studies evaluating the changes of pancreatic ectopic fat before and after LSG using these modalities.

Another reason for performing antecedent LSG was to reduce the risk of POPF. Several risk factors for POPF after distal pancreatectomy (DP) have been reported. Kawaida et al. reported that BMI was a risk factor for POPF after DP using a triple-row stapler (cutoff value: 25.7 kg/m^2^) [19]. However, pancreas thickness is probably the most studied risk factor. Strong evidence connects increasing pancreas thickness with an increased risk of POPF [20]. Bag et al. also recently clarified that the ratio between pancreas thickness and the diameter of the main pancreatic duct (a wide pancreas with a narrow duct) is a significant predictive factor for clinically relevant POPF (*P* = 0.034) [21]. Thus, since antecedent LSG reduces not only visceral fat tissue but also PV and pancreas thickness, we conclude the intraoperative risk during LSPDP and symptomatic POPF lessened.

Another severe complication after LSPDP is splenic/portal vein thrombosis. Elabbasy et al. reported that there was a lower incidence of splenic infarction (risk ratio = 0.17, 95% confidence interval: 0.09–0.33, *P* < 0.001), gastric varices (risk ratio = 0.20, 95% confidence interval: 0.08–0.49, *P* < 0.001) in LSPDP with splenic vessel preservation compared with Warshaw procedure [22]. In addition, Pendola et al. also reported that incidence of splenic/portal vein thrombosis was significantly higher in DP with splenectomy [23]. In this case, splenic vessel preservation was essential; therefore, Warshaw procedure could not be chosen.

First-stage bariatric procedures are usually performed to reduce body weight and visceral or subcutaneous fat tissue to reduce both surgical difficulty and perioperative complications in several surgical fields including abdominal surgery [24, 25]. Of course, radical treatments for malignant diseases should be prior to bariatric procedures if patients have malignant diseases. However, if coexisting diseases can be conservatively observable, first-stage bariatric procedures can be a reasonable alternative for severely obese patients with low-grade abdominal tumors requiring surgical management [26].

## Conclusions

We have reported an important case in which two-stage surgery comprising LSPDP, following LSG as a metabolic surgery, was performed in a severely obesity patient. First-stage LSG before pancreatectomy in severely obese patients is a potential surgical approach to reduce pancreas thickness and recovery of islet β cell function, thereby reducing the risk of clinically relevant POPF.

## Data Availability

The data sets analyzed during the current study are available from the corresponding author on reasonable request.

## Abbreviations

PV: Pancreas volume
PNET: Pancreatic neuroendocrine tumor
LSG: Laparoscopic sleeve gastrectomy
LSPDP: Laparoscopic spleen-preserving distal pancreatectomy
BMI: Body mass index
POPF: Postoperative pancreatic fistula
T2D: Type 2 diabetes
CT: Computed tomography
EUS-FNA: Endoscopic ultrasonographic fine needle aspiration
LRYGB: Laparoscopic Roux-en Y gastric bypass
LSG/DJB: Laparoscopic sleeve gastrectomy with duodenojejunal bypass
DP: Distal pancreatectomy
